# The Effect of Water Mineralization on the Extraction of Active Compounds from Selected Herbs and on the Antioxidant Properties of the Obtained Brews

**DOI:** 10.3390/foods10061227

**Published:** 2021-05-28

**Authors:** Jakub Wyrostek, Radosław Kowalski

**Affiliations:** Department of Analysis and Evaluation of Food Quality, University of Life Sciences in Lublin, 8 Skromna Street, 20-704 Lublin, Poland; jakubwyrostek@gmail.com

**Keywords:** herbal brews, antioxidant activity, polyphenols, flavonoids, mineral compounds in water

## Abstract

The objective of the study was to analyze the effect of total content of minerals in waters on the effectiveness of extraction of polyphenols and flavonoids and on the antioxidant properties of brews from leaves of green and black tea, leaves of peppermint, chamomile inflorescences, leaves of sage, and flowers of lavender. The process of brewing was conducted at an initial temperature of 95 °C, under cover, for 15 min, with the use of 10 waters differing in terms of mineral components. The content of total polyphenols and total flavonoids was determined in the brews obtained. The antioxidant properties were assayed with the use of stable DPPH radicals and Ferric Reducing Antioxidant Power (FRAP) reagent. A significant effect of water mineralization on the extraction of phenolic compounds and on the antioxidant properties of the brews obtained was observed. In the majority of cases, the highest concentrations of polyphenols and flavonoids were obtained in brews prepared with the use of deionized water and waters with medium levels of mineralization. It was also demonstrated that there was a significant reduction of antioxidant properties in brews prepared with the water that had the highest level of mineralization (2896 ppm), at 70% and 93%, respectively, for green and black teas.

## 1. Introduction

Water is an important component in the human diet as it performs a number of various functions which are essential for correct functioning of the organism. It is used for the preparation of various foods and dishes, including drinks, among which brews of tea, coffee or herbs occupy a significant position [1]. The composition of waters available on the market and the level of their mineralization have an impact on the level of their importance as a source of valuable minerals. Mineral waters are underground waters differing from the commonly available mains water in the absence of contaminants and a stable content of minerals. Depending on the concentration of individual elements, such waters can have a lower or higher level of mineralization. Healing, high mineralized waters are characterized by a high level of dissolved salts and even gases which are responsible for their specific pro-health properties. An adequate supply of minerals, including calcium and magnesium, is very important for proper functioning development and functioning of the human body, because they play important physiological roles, e.g., they are the material for bones, they affect the course of metabolic processes and regulation of water and electrolyte balance [2]. Many studies have shown that the deficiency of these nutrients in diets increases the probability of many diseases [3,4].

We classified waters as: high-mineralized waters—containing more than 1500 mg of mineral components in 1 liter of water; medium-mineralized—containing from 500 to 1500 mg of mineral components in liter of water; low-mineralized waters—containing maximum 500 mg of mineral components in 1 liter of water; and very low mineralized waters—with a maximum 50 mg content of mineral components in 1 L of water [5].

In recent years one can observe a growth of the market of bottled water, and consequently an increase of the consumption of such waters. The cause of the increased consumption of bottled water lies in the change of the feeding habits of the consumers. More and more frequently consumers reach for bottled water. This undoubtedly results from the increasing focus on healthy and active lifestyles [6]. Spring waters with low and very-low levels of mineralization are more and more often used for the preparation of brews of tea, coffee and herbs, and for the preparation of food, e.g., for infants. For water to have a beneficial effect on the human organism, it must be characterized by bacteriological and chemical purity, and by a suitable mineral composition, thanks to which it will have positive physiological effects [7]. There has also been an increase of interest in natural medicine, and thus also in herbs in various forms, e.g., herbal teas, diet supplements and medicines. Among the known biologically active compounds present in herbs, special importance is attributed to polyphenols, among which one should also mention flavonoids and other components, e.g., from the group of volatile compounds, such as essential oils which impart the characteristic and specific aroma to herbs and to products derived from them. These active substances perform a number of functions in the human organism, e.g., provide protection against unfavorable effects of UV radiation, and they are natural antioxidants, and pigments [8]. Currently, many studies show that polyphenols have potent activity against a broad spectrum of animal and plant microbes [9,10]. The biological activity of polyphenols largely depends on their bioavailability, in vivo is very low and varies for each individual person [11]. Many of those compounds cannot be synthesized by the human organism, so their supply with food has, among other things, a preventative function, especially in the protection against free radicals [12]. Currently, a lot of attention is paid to the methods of extraction in the field of the green chemistry, as they can reduce or even eliminate the use of dangerous substances, and reduce the costs of neutralization of solvent residues [13]. 

It is worthwhile to take a closer look at herbal brews which can be easily prepared at home, without any need for specialist equipment. Taking into account the quality and safety requirements that must be met by waters with the status of drinking or consumable waters, mains water included, such waters are most frequently used for the preparation of brews. However, nowadays one can observe a trend related with the use of suitable bottled waters for that purpose. This trend results from a variety of determinants, among which one should enumerate the higher quality of bottled waters, relative to mains or tap water. The consumers notice that brews prepared with the use of bottled waters are characterized by more desirable sensory properties, including aroma, compared to brews based on tap water. It would be an interesting approach consisting in the use of mineral waters for the preparation of brews that would combine the pro-health properties resulting both from the presence of biologically active components from the group of secondary metabolites, extracted from herbs, and the presence of mineral components characteristic for a given kind of water. Studies conducted earlier indicate that mineral compounds present in waters, brewing temperature, and pH, have a significant impact on the content of biologically active substances in herbal brews [14,15,16,17]. The composition of tap water and of waters with a high level of mineralization includes large amounts of dissolved mineral salts, especially calcium and magnesium salts, and of hydrogen carbonate ions, which may inhibit the process of extraction and enter into reactions with polyphenolic compounds contained in the herbal raw material, and with pectins present in the cell walls [18,19], reducing the extraction of organic and inorganic compounds and also lowering the antioxidant properties of the brew [20]. In addition, extracts of phenolic compounds from plants can contain various contaminants and interfering substances [21]. The pH is also an important factor in the extraction of phenolics usually a low pH value of the extraction solution can prevent the oxidation of phenolics, though they can also be eliminated through chelation with metal ions [22]. Catechins present in the brew are relatively stable in an acidic environment, but in an alkaline environment they become highly unstable and easily degradable [23]. Therefore, the objective of the study presented herein was to analyze the effect of the level of water mineralization on its capacity for the extraction of polyphenols and flavonoids from selected herbs. The antioxidant properties of the extracts obtained were also analyzed. The addressed subject-matter relates to an innovative approach in the area of preparation of herbal brews that would combine health-promoting properties resulting from the joint presence of biologically active components and mineral components in the brews.

## 2. Materials and Methods

### 2.1. Experimental Material

The plant material used in the experiment was the following: flowers of narrow leaf lavender *Lavandulaangustifolia* L. (Flos, Poland), leaves of common sage *Salvia officinalis* L. (Flos, Poland), leaves of peppermint *Menthapiperita* L. (Flos, Poland); inflorescentia of chamomile *Chamomilla recutita* L. (Flos, Poland), black and green tea leaves *Camellia sinensis* (L.) Kuntze (Big Active, Herbapol-Lublin, Poland). The herbs, originating from a single production batch, were purchased from a pharmacy. The plant material was standardized through pooling together and fragmentation to a homogeneous fraction (aperture size 5.6 mm) with the use of a laboratory analytical mill type A11 Basic (IKA-Werke GmbH, Staufen, Germany), following which, using a moisture analyzer type WPS 50 SX (RADWAG, Radom, Poland), the moisture content of every product was determined in the aspect of conversion to dry matter.

**Figure 1:**
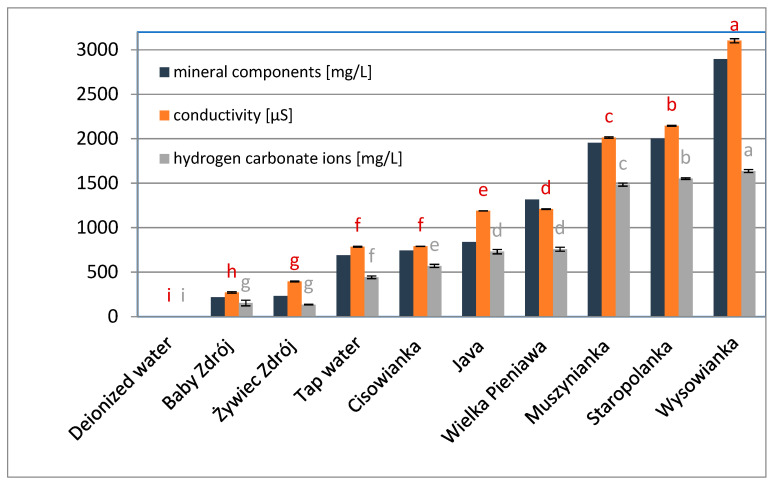
Conductivity, total content of mineral components and hydrogen carbonate ions in selected waters. Data are shown as mean  ±  SD. Different letters (a, b, c, etc.) show a significant difference with *p* < 0.05. The differences relate to each group of compounds separately.

**Figure 2:**
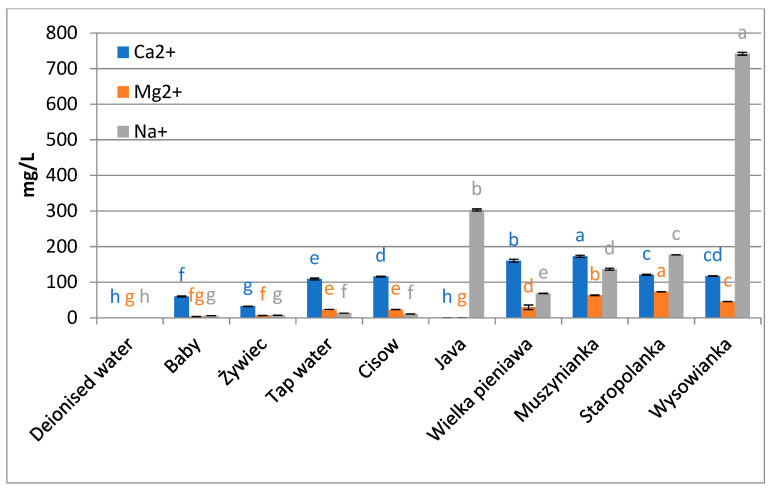
Content of main metal ions in waters used in the experiment. Data are shown as mean  ±  SD. Different letters (a, b, c, etc.) show a significant difference with *p* < 0.05. The differences relate to each ion separately.

**Figure 3:**
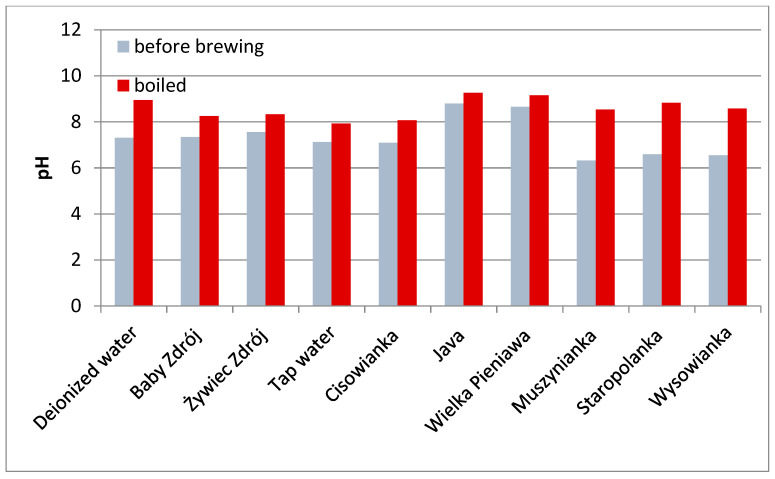
pH of the waters used for the experiment.

The following mineral water brands were used in the study: “Baby Zdrój” (Aqua East Polska), “ŻywiecZdrój” (ŻywiecZdrój), „Cisowianka” (NałęczówZdrój), “Java” (JAVA), tap water (Water supply station Sławinek, Wodna 2, 20-400 Lublin), “WielkaPieniawa” (UzdrowiskaKłodzkie—Grupa PGU), “Muszynianka” (Muszynianka), “Staropolanka” (UzdrowiskaKłodzkie—Grupa PGU), “Wysowianka” (UzdrowiskoWysowa), and as the reference—deionized water (deionization system HLP 5, Hydrolab) (Figure 1, Figure 2 and Figure 3).The total content of mineral components expressed in mg/L was declared by the producers, for comparison, the conductivity and pH of each water was measured using the CX-505 multifunction meter (Elmetron, Zabrze, Poland), moreover the content of hydrogen carbonate ions and Mg^2+^, Ca^2+^, Na^+^ ions was determined.

### 2.2. Carbonate Ions Titration

The content of carbonate ions in the water samples was determined by acid-base titration [24,25]. For this purpose, 100 mL of water were measured into a 250 mL flask, add 2–3 drops of 0.1% methyl orange and titrate with 0.05 M HCl solution until the indicator changes color from yellow to orange. The content of bicarbonates in the solution (in milligrams) was calculated from the formula:

C = V × k × 1000/V_0_

where: V—volume of HCl used for titration of the tested sample (mL);

k—titer of HCl solution in relation to HCO_3_^−^ ions (3.05 mg HCO_3_^−^/mL);
V_0_—volume of the water sample taken for the determination (mL).

### 2.3. Determination of Calcium, Magnesium and Sodium Ions Using Atomic Absorption Spectroscopy (AAS) with Flame Atomization

The concentration of Ca, Mg and Na ions was determined by atomic absorption spectroscopy (AAS) method using a spectrometer SpectrAA 280 FS flame atomization spectrometer (Varian, Mulgrave, Australia) equipped with an automatic standard diluent, SIPS samples, deuterium lamp, air-acetylene burner, hollow cathode lamp for each element. Parameters of analysis:

wavelength—Ca (422.7 nm), Mg (202.6 nm), Na (589.0 nm); 

air flow 10 L/min; 

slit width 0.5 nm (Ca); 

0.2 nm (Na);

nm (Mg);

lamp current 4 mA (Mg), 10 mA (Ca).

Certified single-element standard solutions (1000 mg/L) used to prepare calibration curve were of highest purity grade (99.999%) and were purchased from Ultra Scientific (North Kingstown, RI, USA). The calibration standards for AAS analysis were prepared by diluting certified standard solutions in high purity de-ionized water (deionization system HLP 5, Hydrolab, Straszyn, Poland) purified by reverse osmosis followed by ion-exchange cartridges. 

Table 1 presents the contents of elements in the certified reference material LGC6019.

### 2.4. Preparation of Herbal Infusions

Weighed portions of 5 g of plant material were placed in flasks of 200 mL in volume, and poured over with 100 mL of water, with mineralization level specified by the producer, with initial temperature of 95 °C. Then the flasks were covered with watch glasses. The process of brewing was conducted for 15 min in conformance with the results of an earlier study [26].Then the brews obtained were filtered on paper filters (quantitative paper circles—middle, Filtrak). Since herbal infusions should be consumed within 24 h, brews were used immediately after their preparation for the determination of the concentration of phenolics, flavonoids, and for the analysis of the antioxidant activity. Each brewing was performed in three replicates and analyzed.

### 2.5. Phenolic Compounds Analysis

Determinations of phenolic compounds in the tested macerates were made by spectrophotometric means (λ = 725 nm) according to a modified Singleton and Rossi method [27]. The modification of the method consisted in a proportional change in the volume of individual chemical reagents. The results of the phenols content were expressed in gallic acid equivalents (GAE) (Sigma-Aldrich, St. Louis, MO, USA, ACS reagent ≥ 98.00%). The results were calculated from the equation of the calibration curve prepared for gallic acid standards in the concentration range 10–60 mg L^−1^ (10, 20, 30, 40, 50, 60 mg/L). Each sample, depending on the material of herbal was diluted appropriately to the range of the standard curve. All analyses were performed in triplicate.

### 2.6. Flavonoids Analysis

Determination of flavonoids content in the tested herbal brews was performed by means of spectrophotometry (λ = 510 nm) according to a modified procedure described by Karadeniz et al. [28]. 1 mL of infusion was measured into a 10 mL volumetric flask, 5 mL of redistilled water and 0.3 mL of 5% (*w*/*w*) aqueous sodium nitrate (III) solution were added. The solution was mixed and left for 5 min, added 0.6 mL of 10% (*w*/*w*) aqueous aluminum chloride hexahydrate solution and remixed. After 5 min, it was added 2 mL of 1 M aqueous NaOH solution and made up with redistilled water to the mark. The absorbance of the samples prepared in this manner was measured at 510 nm against the blank. The results of the flavonoid content were expressed in epicatechin equivalents (EE) (Sigma-Aldrich, ACS reagent ≥ 98.00%). The results were calculated from the equation of the calibration curve prepared for epicatechin standards in the concentration range 10–400 mg L^−1^ (10; 50; 100; 150; 200; 250; 300; 400 mg/L). Each sample, depending on the material of herbal was diluted appropriately to the range of the standard curve. All analyses were performed in triplicate.

### 2.7. Free Radical-Scavenging Ability by the Use of a Stable DPPH^•^ Radical

The antioxidant activity was determined according to modified Brand-Williams method et al. with the use of the synthetic radical DPPH (2,2-diphenyl-1-picrylhdrazyl, Sigma) dissolved in ethanol [29,30]. The absorbance of the solutions was measured at the wavelength λ = 517 nm. The obtained solution was diluted so that its absorbance at the wavelength λ = 517 nm was approximately 0.9. The solution was stored in the dark.

The test sample contained 1.5 mL of the DPPH solution and 20 µL of the herbal brews, the absorbance (A) was measured 30 min after the initiation of the reaction. Each measurement was performed in triplicate and the mean absorbance value (A1) for the solution was calculated.

Inhibition of the DPPH radical by the sample was calculated according to the following formula: inhibition % = 100 (A0 − A1)/A0 where A0 is the absorbance of the control and A1 is the absorbance of the sample. Each sample, depending on the material of herbal was diluted appropriately to the range of the standard curve prepared for TROLOX standards. All analyses were performed in triplicate.

### 2.8. Ferric Reducing Antioxidant Power (FRAP) Assay

The total antioxidant potential of a sample was determined using the ferric reducing ability of plasma FRAP assay [31] as a measure of antioxidant power. Briefly, the FRAP reagent was prepared by mixing acetate buffer (300 mM, pH 3.6), a solution of 10 mM TPTZ in 40 mM HCl, and 20 mM FeCl_3_ at 10:1:1 (*v*/*v*/*v*). The reagent (3000 μL) and brew solutions (100 μL) were added to each sample and mixed thoroughly. The absorbance was taken at 593 nm after 180 min. Standard curve was prepared using different concentrations 100–1000 mM of iron sulfate II. All solutions were used on the day of preparation. FRAP unit determines the ability of the reduction of 1 mM of iron (III) to iron (II). The results were expressed as mM Fe^2+^. Analyses were performed in triplicate on each brews.

### 2.9. Statistical Analysis

Data were analyzed using one way ANOVA followed by Duncan’s test using the SAS statistical system (SAS Version 9.1, SAS Inst., Cary, NC, USA). The significance of all tests was set at *p* ≤ 0.05. Data are shown as mean ± SD, Different letters (a, b, c, …) show a significant difference with *p* < 0.05.

## 3. Results 

Analysis of the results obtained revealed a significant effect of the total content of mineral components in water on the concentration of phenolic compounds in the brews, and on their antioxidant properties in particular. 

### 3.1. Content of Polyphenols and Flavonoids

**Figure 4:**
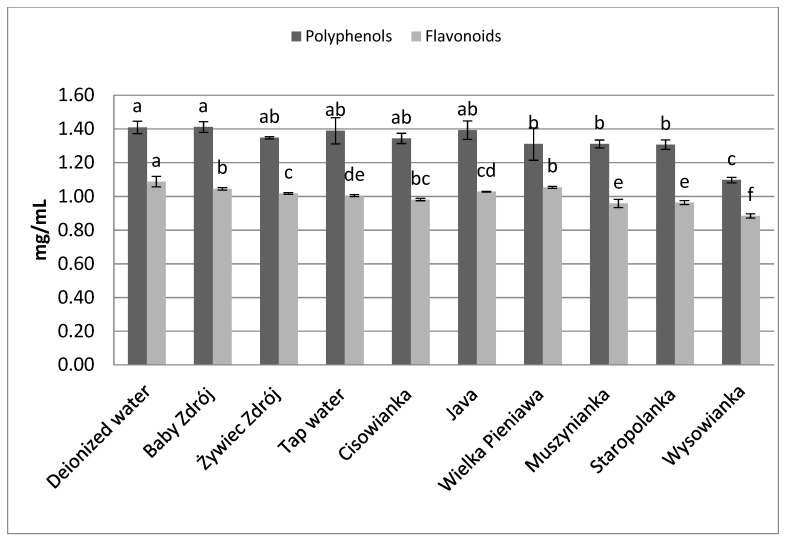
The effect of water mineralization on the extraction of active substances from leaves of peppermint. Data are shown as mean  ±  SD. Different letters (a, b, c, etc.) show a significant difference with *p* < 0.05. The differences relate to each group of compounds separately.

In the case of leaves of peppermint (Figure 4), in brews prepared on the basis of low-mineralized waters higher concentrations of polyphenols and flavonoids were assayed, compared to brews prepared with the use of high-mineralized waters. Statistically the highest concentration of polyphenols was noted in the case of brews prepared with the use of deionized water, and those prepared with waters with mineralization level below 1000 mg/L—from 1.39 to 1.41 mg GAE/mL. Analysis of the content of flavonoids in the brews prepared from leaves of peppermint revealed that the highest concentration of those components was characteristic of the brew prepared with the use of deionized water, at the level of 1.09 mg EE/mL, which was statistically significantly higher than the concentration of flavonoids in brews prepared with the use of the other waters, containing mineral components in their composition. 

**Figure 5:**
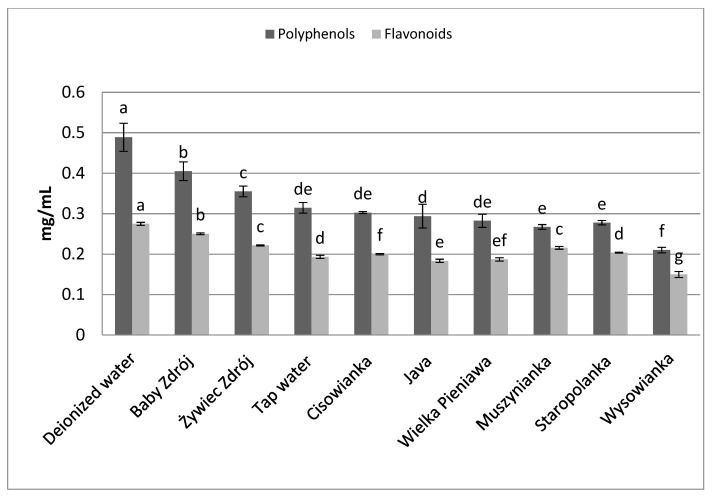
The effect of water mineralization on the extraction of active substances from chamomile inflorescences. Data are shown as mean  ±  SD. Different letters (a, b, c, etc.) show a significant difference with *p* < 0.05. The differences relate to each group of compounds separately.

The brew from chamomile inflorescences (Figure 5) prepared with the use of deionized water was characterized by the highest concentration of polyphenols—0.49 mg GAE/mL, followed by the brew prepared with the use of the water “Baby Zdrój” (with total microelement content of 216 mg/L)—0.40 mg GAE/mL. The highest concentration of flavonoids was assayed in the brew prepared with the use of deionized water—0.27 mg EE/mL. The lowest concentrations of polyphenols and flavonoids were noted for chamomile inflorescence brews prepared with the use of the water “Wysowianka” (characterized by a very high level of mineralization—2896 mg/L), at 0.21 mg GAE/mL and 0.15 mg EE/mL, respectively.

**Figure 6:**
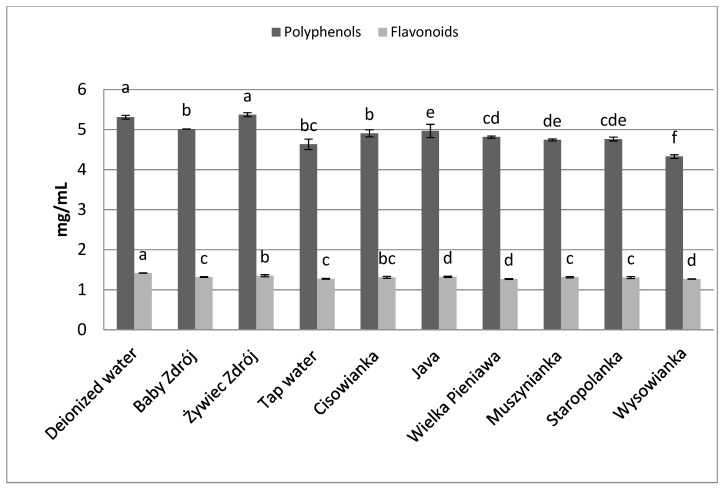
The effect of water mineralization on the extraction of active substances from black tea leaves. Data are shown as mean  ±  SD. Different letters (a, b, c, etc.) show a significant difference with *p* < 0.05. The differences relate to each group of compounds separately.

**Figure 7:**
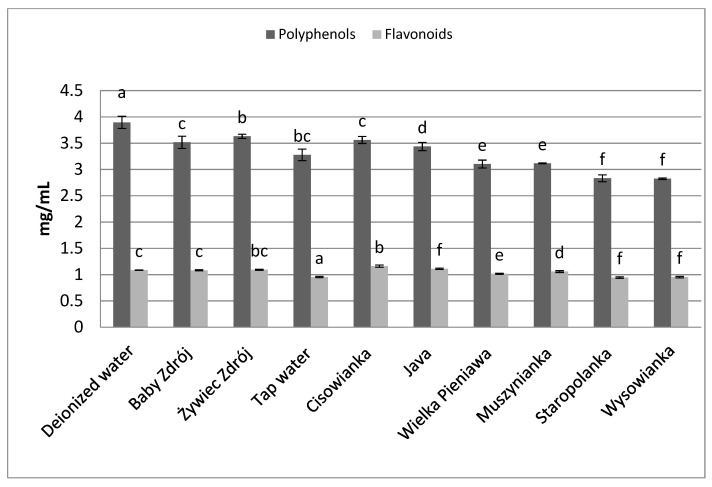
The effect of water mineralization on the extraction of active substances from green tea leaves. Data are shown as mean  ±  SD. Different letters (a, b, c, etc.) show a significant difference with *p* < 0.05. The differences relate to each group of compounds separately.

**Figure 8:**
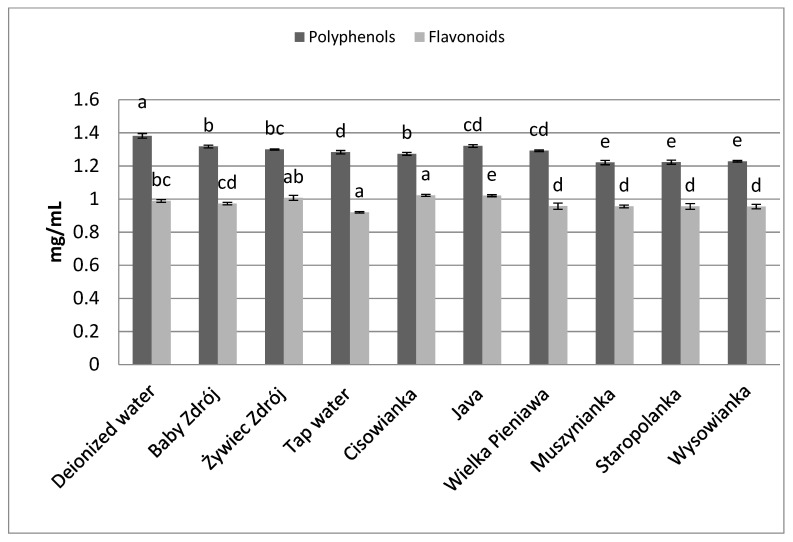
The effect of water mineralization on the extraction of active substances from leaves of sage. Data are shown as mean  ±  SD. Different letters (a, b, c, etc.) show a significant difference with *p* < 0.05. The differences relate to each group of compounds separately.

**Figure 9:**
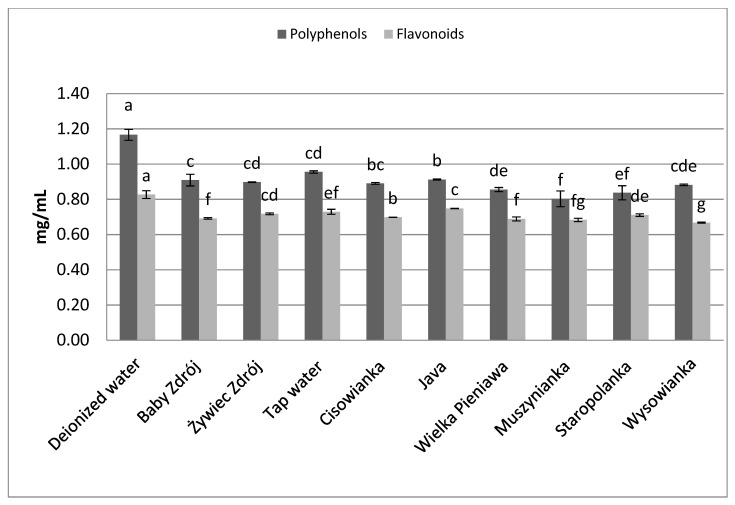
The effect of water mineralization on the extraction of active substances from inflorescences of lavender. Data are shown as mean  ±  SD. Different letters (a, b, c, etc.) show a significant difference with *p* < 0.05. The differences relate to each group of compounds separately.

Similar relations were noted in the case of the other herbs as well as black tea (Figure 6) and green tea (Figure 7). The highest concentrations of polyphenols were assayed in brews prepared with the use of deionized water, and significantly lower in the case of waters with a high level of mineralization. The concentrations of polyphenols and flavonoids in green tea brews were assayed in the range of 2.82–3.89 mg GAE/mL and 0.95–1.09 mg EE/mL, respectively, and in the case of black tea—4.33 mg GAE/mL–5.31 mg EE/mL and 1.27–1.42 mg EE/mL. Identical relationships were noted in the case of brews from leaves of sage (Figure 8) and inflorescences of lavender (Figure 9), for which the highest concentrations of the analyzed components were noted in brews prepared with the use of deionized water, followed by brews based on low-mineralized water and tap water with mineralization level below 1000 mg/L.

### 3.2. Antioxidant Activity

Evaluation of the antioxidant capacity of herbal infusions is not an easy task, as many methods can be used to determine it, and the research material, analytical methods and concentrations used may influence the estimated activity. In this study, two methods of determining the antioxidant activity were used, i.e., the evaluation of the DPPH radical scavenging ability and the FRAP iron ions reduction ability. These methods are most often used in the analysis of antioxidant activity in order to assess the suitability of the studied raw materials as a source of biologically active compounds [32].

It was observed in the experiment that peppermint brews were characterized by an increase of antioxidant properties that was inversely proportional to the content of mineral components, in conversion to mM of Trolox, i.e., from 8.71 to 10.78 mM, depending on the kind water used for the brewing. Statistically the lowest concentration of Trolox was noted in the case of waters with mineralization level above 1954 mg/L. The strongest decrease of antioxidant properties, converted to Trolox, related to the use of highly mineralized waters, was noted in the case of brews from black and green tea. Analyzing tea brews prepared with the use of water with the highest level of mineralization, a decrease was noted in the antioxidant properties, by a maximum of 93.49% in the case of black tea brew, and by 70.32% in the case of green tea brew, relative to brews prepared with the use of deionized water (Table 2 and Table 3).

In the case of the FRAP method, a similar dependence was observed compared to the method using the DPPH radical for chamomile brews (14.37–19.60 mM Fe^2+^), lavender (33.54–46.45 mM Fe^2+^) and green tea (48.17–64, 78 mM Fe^2+^). The increase in antioxidant properties was inversely proportional to the content of minerals in the water used. The lowest antioxidant properties in the case of peppermint, sage, chamomile and green tea brews were recorded for water with the highest mineralization. In addition, the highest decrease in the antioxidant properties in relation to deionized water were noted in the case of sage brews (−31.12%), green tea (−25.64%) and chamomile infusions (−25.20%) (Table 4 and Table 5).

### 3.3. pH Value of Brews

The pH of the waters used in the experiment before the brewing process ranged from 6.32 (Muszynianka) to 8.79 (Java) with no correlation to their overall mineralization. After boiling, all the waters were characterized by an increased pH value, the lowest value was recorded for tap water (7.92), and the highest for Java (9.2) (Figure 3). pH in the obtained herbal brews correlated directly with the content of active compounds and the total mineralization of the water used. Regardless of the initial pH of the boiled water, the lowest pH was recorded for brews with the highest concentration of active compounds prepared in waters with the lowest mineralization. The pH values of the brews were in the ranges: mint—6.55–7.46, chamomile—5.52–7.27, black tea—5.03–6.93, green tea—5.48–7.06, sage—6.24–7.36, and lavender—5.47–7.23 (Table 6).

## 4. Discussion

The high-mineralized waters used in the experiment for the preparation of the brews were characterized also by a high content of calcium ions—from 117 mg/L for the water “Wysowianka”to173 mg/L for the water “Muszynianka”, with a simultaneously very high level of hydrogen carbonate ions—from 1550 mg/L for “Staropolanka” to 1636 mg/L in the case of “Wysowianka”. Calcium ions contained in water react with pectin occurring in plane cell walls, forming a kind of film which has gelling properties. A distinct effect of the water used for brewing on the concentration of compounds in the brew was also observed by Xu et al. [33], analyzing the concentrations of catechins. The cited authors report that the total concentration of catechins in brews prepared with the use of mineral water and tap water is statistically significantly lower compared to brews prepared with the use of pure water and mountain spring water [19]. This proves that water containing a high concentration of minerals can reduce the efficiency of extraction. 

The obtained statistically lower concentrations of biologically active compounds from the group of polyphenolics and flavonoids in brews prepared with the use of waters with high levels of mineralization, compared to brews obtained with the use of waters with lower mineralization levels, correspond with the results obtained by other authors who indicate that during the extraction of those components from tea *Camelia sinensis* (L.) Kuntze so-called “tea cream” can be formed, i.e., a sediment that forms during the cooling down of tea brew as a result of complexation of caffeine with theaflavins or thearubigins [20]. This phenomenon is controlled by a number of parameters, such as pH and brewing temperature [34], and its appearance is facilitated by a high content of calcium ions [35]. The sediment appears only in brews prepared with the use of hard water and it is formed as a result of oxidation of organic compounds, caused by the presence of calcium carbonate, which causes a reduction of the efficiency of extraction both in relation to organic and inorganic compounds, but is also reflected in a reduction of antioxidant properties of herbal brews. The mechanism of extraction of organic and inorganic compounds from tea leaves has been described by Spiro et al. [18]: first, water is absorbed by leaves, and then elements and molecules diffuse from tea leaves to the brew. During the process of brewing in strongly mineralized water, calcium absorption by leaves can take place at the first stage, and the calcium may undergo complexation by pectins present in the cell walls. Apart from calcium ions, also other bivalent metal ions display similar properties, e.g., magnesium ions which can also occur at significant levels both in tap water and in bottled waters, contributing to partial retention of polyphenols in tea residues [36] and to the formation of “tea cream” and “head” [37]. Chen et al. [38] report that also iron ions Fe (II), Fe (III) and copper ions Cu (II), at concentrations of 20 ppm and 5 ppm, respectively, and calcium ions at a concentration of 200 ppm, can significantly reduce the concentration of polyphenols in tea brews. Additionally, Samsonowicz and Regulska [39] demonstrated that the presence of, e.g., calcium and magnesium ions modifies the concentration of polyphenolic compounds assayed with the spectrophotometric method. Metal cations can form complexes with phenolic compounds or they can activate certain functional groups, which may affect the measured concentration of those components and the antioxidant activity of extracts. In our study, the high-mineralized waters used in the process of brewing contained calcium ions at concentrations from 117 to 173 ppm, which also caused a decrease on extraction efficiency and indicates an effect of high levels of those ions on the extraction of active compounds from herbs.

The pH also affects the extraction of polyphenols from plant material. Chethan and Malleshi [22] report that the low pH value of the extraction solution may be a factor preventing phenol oxidation. Similarly, Zeng et al. [40] observed that a more acidic pH stabilized the concentration of polyphenols in infusions. The process of boiling the water resulted in an increase in the pH value. However, after the brewing process, tea and herbal infusions with a lower pH value were obtained, which was also observed by other authors [41].

Comparing the results of antioxidant activity, it can be clearly stated that the results obtained using the FRAP method are higher than those obtained using DPPH radical. These differences may be related to the fact that the oxidative processes in biological systems are complex and closely related to the presence of various active biological compounds, including phenolic compounds and flavonoids [42]. In the research of Hajdari et al. [43] the authors also observed that the determined antioxidant activity of methanol extracts of beech tree is higher using the FRAP method compared to the use of the synthetic DPPH radical. The authors showed that the sum of flavonoids in the extracts was significantly correlated with FRAP. In studies by Assa et al. [44] also showed that the content of phenolic compounds in nutmeg extracts is strongly correlated with ferric reducing antioxidant power. The high content of these compounds increased the ability to reduce iron ions. In turn, in the work of Hinnenburg et al. [45] the authors showed that the content of phenolic compounds and FRAP were strongly correlated (R = 0.887) in alcoholic extracts of basil, parsley, juniper, fennel, caraway, cardamom and ginger. Additionally, a similar relationship was shown by other authors [46,47]. In the studies of Hidalgo et al. [48] showed a tendency of flavonoids to combine into various mixtures/interact with other compounds during which hydrogen bonds are formed between individual flavonoids, reducing access to hydroxyl groups, which in turn may reduce the interaction with the synthetic DPPH radical and ultimately lower the results of the antioxidant activity of this method. The results of the same authors also indicate possible interactions of flavonoids increasing antioxidant activity determined by the FRAP method. Based on the literature data, we can conclude that the flavonoids present in herbs/food can interact and influence the total antioxidant capacity. It can also be concluded that there are synergistic and antagonistic effects of individual phenolic compounds when measuring antioxidant properties. Although both DPPH and FRAP tests are used to determine antioxidant activity, they will not always give similar results [49].

## 5. Conclusions

Summing up the above research results, one can conclude that the level of mineralization of water used for the preparation of popular tea or herbal brews has an impact on the process of extraction of bioactive components such as polyphenols and flavonoids, and on the antioxidant activity of such brews. It is worth emphasizing that suitable choice of the level of mineralization of water used in the process of brewing allows to obtain a brew that will combine health-promoting properties resulting both from the presence of mineral components and of biologically active components of plant origin. Therefore, it is preferable to use waters with low or medium levels of mineralization for the process of brewing, and with simultaneous low level of calcium ions which inhibit the extraction of active components from tea and herbs. 

## Figures and Tables

**Table 1:** The content of elements in the certified reference material LGC6019 and validation parameters obtained during analysis.

Element	Certified Reference Material Analysis		Validation Parameters			
	The Result Declared by the Manufacturer	The Result Obtained in Own Research	Limit of Detection	Limit of Quantification	Precision (%)	Uncertainty (%)
Ca (mg/L)	109	108	0.05	0.11	0.4	18
Mg (mg/L)	4.62	4.57	0.06	0.12	0.7	22
Na (mg/L)	24.7	24.4	0.05	0.10	0.6	21

**Table 2:** Percentage decrease of antioxidant activity (converted to mM of Trolox) of peppermint, chamomile and black tea brews prepared with the use of mineral waters relative to the brew prepared with the use of deionized water.

	Peppermint		Camomile		Black Tea	
	Trolox mM	Difference %	Trolox mM	Difference %	Trolox mM	Difference %
Deionised water	10.78 ± 0.16 a ^1^	0.00	2.30 ± 0.10 a	0.00	15.67 ± 1.51 a	0.00
Baby Zdrój	10.02 ± 0.69 bcd	−7.04	2.15 ± 0.07 a	−6.83	12.82 ± 1.84 b	−18.16
Żywiec	10.19 ± 0.22 abc	−5.52	1.66 ± 0.06 b	−28.16	12.92 ± 0.80 b	−17.57
Tap water	10.62 ± 0.28 ab	−1.46	1.51 ± 0.04 bcd	−34.45	10.31 ± 0.51 c	−34.21
Cisowianka	9.32 ± 0.25 de	−13.58	1.58 ± 0.08 bc	−31.58	10.25 ± 0.72 c	−34.59
Java	9.90 ± 0.13 cd	−8.18	1.50 ± 0.03 bcd	−35.01	8.49 ± 0.26 d	−45.83
Wielka pieniawa	9.98 ± 0.64 bcd	−7.41	1.61 ± 0.11 b	−30.11	6.78 ± 0.77 d	−56.71
Muszynianka	9.39 ± 0.25 de	−12.95	1.36 ± 0.20 d	−40.99	1.20 ± 0.76 f	−92.35
Staropolanka	9.38 ± 0.53 de	−13.00	1.39 ± 0.21 cd	−39.87	3.91 ± 0.75 e	−75.04
Wysowianka	8.71 ± 0.08 e	−19.26	0.87 ± 0.19 e	−62.44	1.02 ± 0.95 f	−93.49

^1^ Data are shown as mean  ±  SD. Different letters (a, b, c, etc.) show a significant difference with *p* < 0.05. The differences relate to each group of brews separately.

**Table 3:** Percentage decrease of antioxidant activity (converted to mM of Trolox) of green tea, sage and lavender brews prepared with the use of mineral waters relative to the brew prepared with the use of deionized water.

	Green Tea		Sage		Lavender	
Tested Water	Trolox mM	Difference %	Trolox mM	Difference %	Trolox mM	Difference %
Deionised water	15.56 ± 0.49 a ^1^	0.00	11.29 ± 0.16 a	0.00	7.96 ± 0.08 a	0.00
Baby Zdrój	14.03 ± 0.31 b	−9.82	9.09 ± 0.20 c	−19.49	7.08 ± 0.49 b	−11.00
Żywiec	13.06 ± 0.77 c	−16.05	10.25 ± 0.30 b	−9.16	7.37 ± 0.35 ab	−7.46
Tap water	12.33 ± 0.56 c	−20.75	9.74 ± 0.34 bc	−13.72	7.27 ± 0.24 b	−8.65
Cisowianka	13.82 ± 0.38 ab	−11.15	9.69 ± 0.49 bc	−14.17	5.96 ± 0.50 c	−25.09
Java	10.04 ± 0.52 d	−35.49	10.30 ± 0.21 b	−8.75	6.43 ± 0.61 c	−19.26
Wielka pieniawa	9.92 ± 0.74 d	−36.21	7.64 ± 0.69 d	−32.27	4.85 ± 0.20 d	−39.06
Muszynianka	7.98 ± 0.68 e	−48.70	8.19 ± 0.31 d	−27.44	4.53 ± 0.44 d	−43.06
Staropolanka	7.38 ± 0.20 e	−52.53	7.77 ± 0.08 d	−31.19	4.98 ± 0.20 d	−37.46
Wysowianka	4.62 ± 0.23 f	−70.32	7.82 ± 0.55 d	−30.68	4.32 ± 0.16 d	−45.76

^1^ Data are shown as mean  ±  SD. Different letters (a, b, c, etc.) show a significant difference with *p* < 0.05. The differences relate to each group of brews separately.

**Table 4:** Percentage decrease of Ferric Reducing Antioxidant Power (FRAP) (converted to mM Fe^2+)^ of peppermint, chamomile and black tea brews prepared with the use of mineral waters relative to the brew prepared with the use of deionized water.

	Peppermint		Camomile		Black Tea	
	Fe^2+^ mM	Difference %	Fe^2+^ mM	Difference %	Fe^2+^ mM	Difference %
Deionised water	44.51 ± 2.24 cd ^1^	0.00	19.21 ± 0.36 a	0.00	63.67 ± 1.52 de	0.00
Baby Zdrój	44.62 ± 2.62 cd	0.25	18.56 ± 0.52 a	−3.38	61.30 ± 1.20 e	−3.72
Żywiec	38.06 ± 3.98 fg	−14.49	15.86 ± 1.84 bc	−17.44	67.05 ± 0.14 bc	5.31
Tap water	40.24 ± 1.15 ef	−9.59	19.6 ± 0.17 a	8.43	70.62 ± 1.28 a	10.92
Cisowianka	42.58 ± 0.96 de	−4.34	16.32 ± 0.34 b	−15.04	69.06 ± 1.56 ab	8.47
Java	34.77 ± 1.41 g	−21.88	16.10 ±0.93 b	−16.19	69.55± 1.06 ab	9.24
Wielka pieniawa	50.29 ± 2.84 b	12.99	16.07 ± 0.71 b	−16.35	69.79 ± 3.79 ab	9.61
Muszynianka	54.79 ± 2.90 a	23.10	15.63 ± 0.51 bc	−18.64	70.17 ± 0.55 a	10.21
Staropolanka	47.24 ± 1.87 bc	6.13	16.68 ± 0.61 b	−13.17	67.72 ± 0.84 abc	6.36
Wysowianka	40.95 ± 01.90 ef	−8.00	14.37 ± 0.71 c	−25.20	65.92 ± 1.98 cd	3.53

^1^ Data are shown as mean  ±  SD. Different letters (a, b, c, etc.) show a significant difference with *p* < 0.05. The differences relate to each group of brews separately.

**Table 5:** Percentage decrease of Ferric Reducing Antioxidant Power (FRAP) (converted to mM Fe^2+^) of green tea, sage and lavender brews prepared with the use of mineral waters relative to the brew prepared with the use of deionized water.

	Green Tea		Sage		Lavender	
Tested Water	Fe^2+^ mM	Difference %	Fe^2+^ mM	Difference %	Fe^2+^ mM	Difference %
Deionised water	64.78 ± 0.29 a ^1^	0.00	67.67 ± 0.75 bc	0.00	46.45 ± 0.23 a	0.00
Baby Zdrój	51.01 ± 0.71 d	−21.26	65.71 ± 0.37 cd	−2.90	35.00± 2.34 c	−24.65
Żywiec	57.26 ± 1.71 bc	−11.61	65.31 ± 3.15 cd	−3.49	34.17 ± 1.42 c	−26.44
Tap water	58.07 ± 2.17 b	−10.36	70.31 ± 2.09 b	3.90	33.54 ± 1.47 c	−27.79
Cisowianka	56.29 ± 0.52 bc	−13.11	62.50 ± 1.31 d	−7.64	40.84 ± 1.58 b	−12.08
Java	51.79 ± 2.50 d	−20.05	66.22 ± 4.40 c	−2.14	33.72 ± 0.96 c	−27.41
Wielka pieniawa	55.01 ± 2.15 c	−15.08	57.15 ± 0.42 e	−15.55	35.68 ± 2.87 c	−23.19
Muszynianka	56.62 ± 0.36 bc	−12.60	68.01 ± 3.12 bc	0.50	41.74 ± 0.76 b	−10.14
Staropolanka	55.01 ± 0.80 c	−15.08	76.32 ± 0.69 a	12.78	42.43 ± 0.45 b	−8.65
Wysowianka	48.17 ± 1.34 e	−25.64	46.61 ± 0.22 f	−31.12	40.54 ± 1.09 b	−12.72

^1^ Data are shown as mean  ±  SD. Different letters (a, b, c, etc.) show a significant difference with *p* < 0.05. The differences relate to each group of brews separately.

**Table 6:** pH values of the herbal infusions prepared with the water used in the experiment.

	Peppermint	Camomile	Black Tea	Green Tea	Sage	Lavender
Deionised water	6.55	5.52	5.03	5.48	6.24	5.47
Baby Zdrój	6.8	5.55	5.49	5.55	6.7	5.67
Żywiec	6.8	6.05	5.52	6.67	6.72	5.94
Tap water	6.96	6.43	6.12	6.45	6.98	6.01
Cisowianka	7.06	6.65	6.52	6.61	7.04	6.56
Java	7.25	6.78	6.61	6.94	7.15	7.01
Wielka pieniawa	7.12	6.84	6.6	6.82	7.12	6.98
Muszynianka	7.44	7.12	6.69	6.95	7.12	7.01
Staropolanka	7.25	7.07	6.7	6.91	7.26	7.05
Wysowianka	7.46	7.27	6.93	7.06	7.36	7.23

## Data Availability

The data used to support the findings of this study are included within the article.
